# Supplementary material for: AffyMAPSDetector: a software tool to characterize Affymetrix GeneChip™ expression arrays with respect to SNPs
Source: BMC Bioinformatics. 2007 Jul 30;8:276. doi: 10.1186/1471-2105-8-276 (PMC1959249; doi:10.1186/1471-2105-8-276)
Supplement: Additional file 13 — AffyMAPSDetector v1 source code. AffyMAPSDetector source code. [file 1471-2105-8-276-S13.zip › AffyMAPSDetector_v1_src/doc/jwf/package-summary.html]

jwf


|  |  |  |  |
| --- | --- | --- | --- |
| **Overview** \| **Package** \| Class \| **Use** \| **Tree** \| **Deprecated** \| **Index** \| **Help** | | |  |
| **PREV PACKAGE**   NEXT PACKAGE | **FRAMES**    **NO FRAMES**     **All Classes** |  |  |


---

## Package jwf

This package contains classes that can be used to create and run a
Wizard.

**See:**
  
          **Description**

|  |  |
| --- | --- |
| **Interface Summary** | |
| **WizardListener** | Implement this interface to be able to receive wizard related events. |

|  |  |
| --- | --- |
| **Class Summary** | |
| **ErrorMessageBox** | Displays a list of error messages and blocks until ok is pressed. |
| **NullWizardPanel** | An implementation of the base class used for implementing a panel that is displayed in a Wizard. |
| **Wizard** | This class controls a wizard. |
| **WizardAdapter** | This class provides means of abreviating work when using the WizardListener allowing the developer to implement only the needed methods |
| **WizardContext** | An instance of this class is maintained by the Wizard. |
| **WizardPanel** | The base class used for implementing a panel that is displayed in a Wizard. |
| **WizardUtils** | Description: This is a general purpose utility class that is a convenient place for wizard-wide utitilites. |

## Package jwf Description

This package contains classes that can be used to create and run a
Wizard.

---


|  |  |  |  |
| --- | --- | --- | --- |
| **Overview** \| **Package** \| Class \| **Use** \| **Tree** \| **Deprecated** \| **Index** \| **Help** | | |  |
| **PREV PACKAGE**   NEXT PACKAGE | **FRAMES**    **NO FRAMES**     **All Classes** |  |  |


---
